# Multi-Objective Optimisation of a Novel Bypass Graft with a Spiral Ridge

**DOI:** 10.3390/bioengineering10040489

**Published:** 2023-04-19

**Authors:** Antonios Xenakis, Andres Ruiz-Soler, Amir Keshmiri

**Affiliations:** 1 School of Engineering, The University of Manchester, Manchester M13 9PL, UK; 2 Department of Cardiothoracic Surgery, Manchester University NHS Foundation Trust, Manchester M13 9WL, UK

**Keywords:** CFD, haemodynamics, spiral flow, multi-objective optimisation, bypass grafts, anastomosis

## Abstract

The low long-term patency of bypass grafts is a major concern for cardiovascular treatments. Unfavourable haemodynamic conditions in the proximity of distal anastomosis are closely related to thrombus creation and lumen lesions. Modern graft designs address this unfavourable haemodynamic environment with the introduction of a helical component in the flow field, either by means of out-of-plane helicity graft geometry or a spiral ridge. While the latter has been found to lack in performance when compared to the out-of-plane helicity designs, recent findings support the idea that the existing spiral ridge grafts can be further improved in performance through optimising relevant design parameters. In the current study, robust multi-objective optimisation techniques are implemented, covering a wide range of possible designs coupled with proven and well validated computational fluid dynamics (CFD) algorithms. It is shown that the final set of suggested design parameters could significantly improve haemodynamic performance and therefore could be used to enhance the design of spiral ridge bypass grafts.

## 1. Introduction

Coronary Artery Disease (CAD) and Peripheral Vascular Disease (PVD) are two of the leading causes of mortality, currently showing an increase of 150% from 1990 to 2013 [1]. Arterial Bypass Grafts (ABG) are one of the leading means to restore a normal perfusion of the affected vessels but are often associated with failures [2] and unsatisfactory long-term performances [3]. Similarly, Arterio-Venous access Grafts (AVGs) used for haemodialysis are suffering low patency because of occurring stenosis [4]. Much research is being performed to reduce failure rates and improve patency rates, especially with the use of Computational Fluid Dynamics (CFD) methods (e.g., [5,6,7,8,9,10]).

Common factors in the low patency ABGs and AVGs are the formation of lumen lesions, such as Intimal Hyperplasia (IH) and thrombosis, which are closely related to haemodynamic factors [11,12]. In addition, intimal hyperplasia/thickening are considered to be the precursor lesions for atherosclerosis in humans, and atherosclerotic lesions have also been shown to develop at sites of pre-existing intimal hyperplasia/thickening [13]. Thus, a significant number of studies have been carried out to improve the patency of bypass grafts by improving the flow environment in the host artery while suggesting improved graft designs, taking into account parameters such as the anastomotic angle, shape of the distal anastomosis, out-of-plane graft, graft-to-host artery diameter ratio, competitive flow, and distance of grafting [12,14].

A recent approach to improving the haemodynamics around distal anastomosis was based on the finding that normal flow conditions in the circulatory system involve a helical component in the flow field induced by the twisting left ventricle during contraction [15]. These observations have been realised in fully operational grafts, with examples such as the ‘SwirlGraft’, developed by Caro et al. [16] at Veryan Medical Ltd. Horsham, UK, and the ‘Spiral Flow Peripheral Vascular Graft’, initially studied by Stonebridge et al. [17,18] and subsequently commercialised by Vascular Flow Technologies. Both of the aforementioned designs induce a helical flow field in the host artery, although they utilise different approaches. The ‘SwirlGraft’ uses an out-of-plane geometry, while the ‘Spiral Flow Peripheral Vascular Graft’ uses a helical internal ridge that adds a spiral component to the flow field. Extensive work has been carried out in the literature to examine the effects of the two designs, with more work concentrated on the out-of-plane graft geometries [19,20,21,22,23,24], while the spiral ridge is less examined [25,26]. The present authors [27,28] have examined both concepts and shown that grafted out-of-plane helicity is significantly more effective than a spiral ridge, while their combination can further enhance the swirling effect in the flow. Moreover, Ruiz-Soler et al. [26] have performed a parametric study that highlighted the possibility of significantly improving the performance of the peripheral spiral ridge bypass grafts by varying a number of key geometrical factors. It was found that the trailing ridge orientation can significantly improve the flow field near the end anastomosis site. Moreover, the height of the elliptical ridge and the pitch of the ridge have been shown to play a less significant role.

CFD has shown to be a powerful and cost-effective tool for investigating the haemodynamic performance of such flow problems as well as other cardiovascular diseases and medical devices, which includes some of the recent work by the present authors [29,30,31,32,33,34,35,36,37,38]. Therefore, in the present study, robust optimisation techniques are coupled with well-validated CFD methods with the aim of finding an ‘optimal’ graft design with the spiral ridge. A wide range of design parameters are considered, taking into account the existing spiral ridge design along with a careful choice of the optimisation criteria. The outcome is a ridge geometry that significantly improves the performance of the currently used design. Moreover, although such optimisation studies are common in other engineering applications (e.g., for CFD applications [39,40,41,42,43]), they have rarely been utilised in biomedical engineering problems (e.g., [44,45,46]). Therefore, the current work also shows the advantages of using goal-driven optimisation studies in the context of biomedical research and their potential impact on the design of new medical devices.

## 2. Optimisation Approach

### 2.1. Introduction

Conducting a multi-objective optimisation for a peripheral bypass graft is a complex process requiring advanced computational and mathematical techniques. In this section, the definition of the problem is presented, focusing on the existing spiral ridge graft designs and the haemodynamics associated with the lesions forming in the proximity of the distal anastomosis. Subsequently, the relevant design parameters are explored. Finally, details of the CFD solver and the optimisation techniques implemented in the present study are provided.

### 2.2. Optimisation Criteria

**Figure 1:**
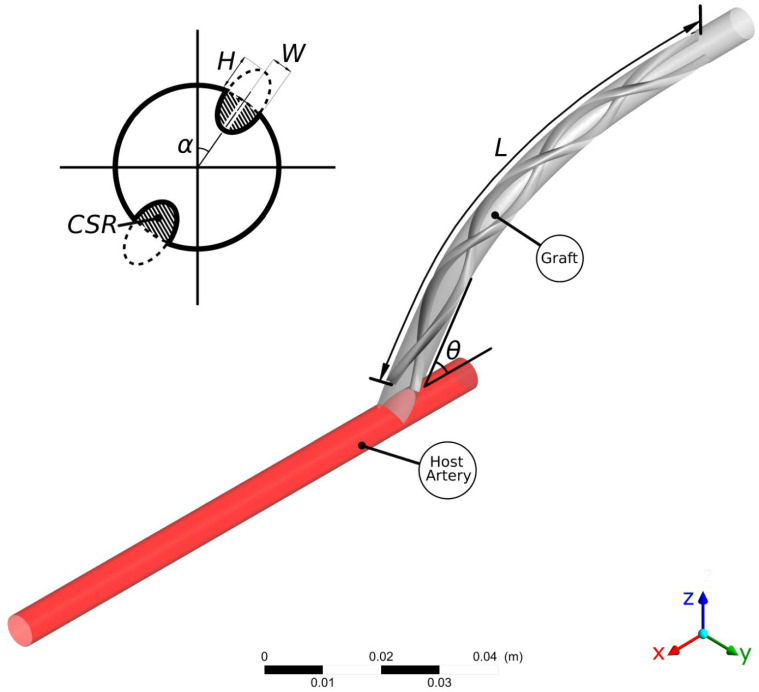
Computational configuration of the graft and host artery for the multi-objective optimisation study, here presented for a case with two ridges: CSR is the occlusion area; H and W are the ridges’ height and width; α is the trailing edge orientation; θ is the anastomosis angle; and L is the ridge length.

In the present work, the design optimisation of the flow characteristics around a distal graft anastomosis is examined. As shown in Figure 1, a peripheral bypass graft with a spiral ridge is anastomosed to a host artery. The internal diameter of both the graft and the host artery is 6 mm. The introduction of a spiral ridge in the graft results in an improvement in the haemodynamic environment in the host artery, which is closely related to graft failure through IH and thrombosis in the regions near the anastomosis. Although the helical flow pattern is considered beneficial [47], it is not yet clear in the literature which haemodynamic conditions are specifically associated with the formation of lesions [48]. In fact, conducting an in vivo study that will highlight the haemodynamic factors involved in lesion formation is still a major challenge [49]. Nevertheless, the existing literature has repeatedly associated local haemodynamic metrics with vascular lesions [11,12,28,50]. It has been shown by a number of authors that low values of Wall Shear Stress (WSS) are a common factor in plaque formation [51,52,53], intimal medial thickness increase [54], and fibroatheroma and intermediate lesion proliferation [55,56]. Moreover, excessive values of WSS may also result in endothelial lesions [25]. There have also been a number of studies that identify the spatial WSS Gradient (WSSG) as a factor that triggers endothelial cell responses [57,58] and thus atherosclerosis.

In light of the above findings, in the present study the haemodynamic optimisation criteria of the spiral-inducing graft will be based on the assumption of high WSS values and more specifically on the minimisation of the areas where WSS is below a certain threshold (i.e., WSS Areas (WSSA) lower than 1 Pa according to [59]) and the minimisation of WSSG within the anastomosis. Moreover, one should also aim to minimise the recirculation areas caused by flow separation, while an increased measure of helicity will be viewed as a potential favourable haemodynamic factor.

### 2.3. Design Parameters

In this study, several different parameters are explored with the aim of covering a wide range of design possibilities. All the design variations examined here would involve one or multiple ridges with an elliptical shape. In order to control the shape and size of the elliptical ridges, the following two dimensionless parameters are defined:

(1) $$\mathrm{Height}\;\mathrm{over}\;\mathrm{Width}\;\mathrm{ratio}\;(\mathrm{HoW})=\frac{Ellipsis\;Height}{Ellipsis\;Width},$$

(2) $$\mathrm{Cross}-\mathrm{Sectional}\;\mathrm{Ratio}\;(\mathrm{CSR})=\frac{A_{r}\times N_{r}}{A_{H}}.$$

HoW is used to control the shape of the ridge cross-section, while CSR defines the area covered by the ridges. In Equation (2), $A_{r}$ and $N_{r}$ are the cross-sectional areas of the ridge and the number of spiral ridge(s), respectively, while $A_{H}$ is the cross-sectional area of the host artery (or the non-occluded graft). In this study, a single ridge design is only considered per case examined. Thus, when multiple ridges are used ($N_{r}>1$) the same design is shared.

Additional design parameters that are examined in this study include the circular orientation of the ridge trailing edge (hereafter referred to as Trailing Edge Orientation—TEO), the ridge length, and pitch. The TEO, which represents the circumferential location of the ridge trailing edge within the graft, just before reaching the anastomosis, is examined in the whole spectrum between $0^{0}{\;\mathrm{and}\;360}^{o}$. Following the work of Ruiz-Soler et al. [26], the orientation of the ridge can be crucial to the performance of the helical graft. The variable that is introduced to control the TEO is α which takes a value in the range of $\alpha \in \left[0,360^{o}/N_{R}\right]$. Therefore, no orientation is examined more than once for the symmetrical designs with $N_{R}>1$. Moreover, a wide range of ridge lengths (*L*) are examined, since the use of excessive ridge length will increase the resistance of the flow within the graft, therefore reducing the efficiency of the design [28]. The effects of the pitch (φ) which is determined as ridge turns per unit length, are also examined. For efficiency, the nominal length ($L_{0}$) is introduced, which refers to the initial ridge length of the ridge, $L_{0}=15\times D=90\;\mathrm{mm}$, so that the pitch φ can now be written as follows:

(3) $$\varphi =Turns/L_{0}.$$

Finally, the anastomosis angle θ between the graft and the host artery is examined. This parameter is not directly related to the graft design and may vary significantly between different suture operations. However, it has been shown previously that θ might be an important factor in the overall graft performance, thus making it a relevant factor to take into account in the present study [12].

### 2.4. Goal-Driven Optimisation

Goal-Driven Optimisation (GDO) is the process of finding solutions that satisfy the objectives while minimising the trade-offs of a given problem [60]. The GDO process is performed by initially generating a set of sample points using a Latin hypercube-based sampling method [61,62]. The sample points are iteratively generated and post-processed by ANSYS DesignXplorer [61,63] so that the sample points are optimally distributed within the design space [62].

The output results for each sample point are calculated with ANSYS CFX using the method presented in Section 2.4. These results are then composed into response surfaces using a non-parametric regression algorithm [64,65]. This allows for any output parameter, *Y*, to be represented by a continuous function in terms of the input samples $X=\{\vec{X_{1}},\vec{X_{2}},\ldots ,\vec{X_{M}}\}$ where $\vec{X_{i}}$ is an *N*-dimensional vector and represents an input set of parameters. Using a kernel map $K(\vec{X_{i}},\vec{X})$, Y can be written as follows:

(4) $$Y=\sum_{i=1}^{N}\left(A_{i}-A_{i}^{*}\right)K\left(\vec{X_{i}},\vec{X}\right)+b,$$

with $A_{i}$ and $A_{i}^{*}$ being the minimum set of Lagrange multipliers that satisfy the system

(5) $$\left\{\begin{array}{c} L=0.5\sum_{i=1}^{N}\sum_{j=1}^{N}\left(A_{i}^{*}-A_{i}\right)\left(A_{j}^{*}-A_{j}\right)K\left(\vec{X_{i}},\vec{X_{j}}\right)+\sum_{i=1}^{N}\left[\varepsilon \left(A_{i}^{*}+A_{i}\right)-y_{i}\left(A_{i}^{*}-A_{i}\right)\right]\\ 0\leq A_{i}^{*}\leq C\\ \sum_{i=1}^{N}\left(A_{i}^{*}-A_{i}\right)=0 \end{array}\right.,$$

with ε being the tolerance from the regression line and C being an arbitrary positive constant. The minimum $A_{i}^{*},A_{i}$ set is found using a quadratic programming optimiser, while the constant b from Equation (4) is obtained by the application of Karush–Kuhn–Tucker conditions [66,67]. For more information on the application of a non-parametric regression algorithm with ANSYS DesignXplorer the reader is referred to [62].

A robust multi-objective evolutionary algorithm based on the Non-dominated Sorted Genetic Algorithm-II (NSGA-II) [62,68] is then used to identify optimal solutions within the continuous representation of the design space, obtained from Equations (4) and (5) The genetic algorithm operates by generating new sets (‘populations’) of possible solutions from the existing ones [69,70] by conducting two main operations, namely ‘crossover’ and ‘mutation’. With crossover, two parent solutions are combined to give a new set of possible solutions (‘offspring’). The logic behind this approach is that an offspring solution should be better than its parents if the optimal characteristics from the two parents are inherited [60,69]. A typical crossover operator linearly combines two parent solutions, $P_{1}$ and $P_{2}$, in order to produce two new offspring, $O_{1}$ and $O_{2}$ in the following form:

(6) $$\left\{\begin{array}{c} O_{1}=a\;P_{1}+\left(1-a\right)P_{2}\\ O_{2}=\left(1-a\right)P_{1}+a\;P_{2} \end{array}\right.,$$

where a is a constant with 0<a<1. With the formulation of Equation (6), a new set of offspring solutions is generated, while with a ‘mutation’ algorithm, some of these results are altered within bounding limits and by using a polynomial distribution function $\left(\delta \right)$ so that local optima are avoided [60,69]. The number of altered points is controlled by the mutation probability, $P_{mut}$, which is of the order of 1–20%, with higher values indicating a higher degree of randomness by the optimisation algorithm. The optimisation algorithm is iteratively executed, producing solutions that are evaluated and classified into ‘Pareto’ and ‘non-Pareto’ solutions. The stopping criterion for this optimisation process is reached when at least 80% of the offspring solutions belong in the Pareto front, which optimally satisfy the improvement criteria discussed in Section 2.2 and presented in Table 1.

### 2.5. Computational Approach

In the present computations, an incompressible laminar flow is assumed, which can be represented by the set of three-dimensional Navier–Stokes equations, where the continuity equation can be written as follows:

(7) ∇·u=0

and the momentum equation,

(8) $$\rho \frac{du}{dt}=-\nabla P+\nabla \cdot \tau ,$$

where ρ is the density of the blood, u is the velocity vector, P is the pressure, and τ is the stress tensor, which is defined as follows:

(9) $$\tau =\mu_{\mathrm{eff}}\left(\left|D\right|\right)D,$$

where D and $\left|D\right|$ are the rate of deformation tensor and its scalar measure, respectively, and $\mu_{\mathrm{eff}}$ is the effective dynamic viscosity of the blood, which is modelled as a Carreau-Yasuda shear-thinning non-Newtonian model [71] as follows:

(10) $$\mu_{eff}=\mu_{\infty }+\frac{\mu_{0}-\mu_{\infty }}{{\left[1+{\left(\lambda \left|D\right|\right)}^{a}\right]}^{\frac{1-n}{a}}},$$

with the fitting parameters $\mu_{0}$, $\mu_{\infty }$, λ, a, and n taking appropriate values for blood simulations as $\mu_{0}=22\times 10^{-3}Pa\cdot s$, $\mu_{\infty }=2.2\times 10^{-3}Pa\cdot s$, λ=0.11s, a=0.644, and n=0.392, respectively [72]. Common values for the blood density are also used, with $\rho =1050\mathrm{kg}/m^{3}\;$ [73,74]. As shown in Equation (8), the external body forces were neglected in this study.

The graft configurations examined here are discretised using tetrahedral meshes with the use of prismatic elements closer to the wall, having a maximum face size of $4\times 10^{-4}\;m$. A mesh independence test has been carried out along with skewness and orthogonality checks for the mesh, which is created using ANSYS Meshing (Version 15.0).

Appropriate boundary conditions were implemented to solve the Navier–Stokes equations. The simulations carried out in this project are based on a constant mass flow rate. A fully developed velocity distribution is applied at the inlet, with an average velocity that corresponds to a Reynolds number *Re* = 570 in the case of a 6 mm diameter graft without ridges. A no-slip boundary condition is applied to all walls, and a rigid wall model is assumed [75,76].

The governing equations were solved numerically by a finite-volume method and the CFD code, ANSYS-CFX, using a fully implicit second-order backward Euler differencing scheme. The convergence criterion (a normalised residual obtained based on the imbalance in the linearized system of discrete equations) was set to 10^−5^ in this study.

## 3. Results and Discussion

### 3.1. Introduction

In this section, the results of the optimisation study are presented. The analysis of the results is made in two separate parts. Firstly, a GDO study is conducted, and five candidate designs are identified. Following an analysis of gains and trade-offs, the final design of the GDO is chosen. Secondly, the optimised design is assessed against a control graft (i.e., a conventional tubular bypass graft configuration with no ridges) and a baseline ridge design as presented in [28] (representing the design used in current practice [17,18]). Important haemodynamic metrics are compared both for steady-state and transient simulations, and the advantages of the proposed design are clearly illustrated.

### 3.2. Goal-Driven Optimisation Study

As was alluded to in Section 2.5, an evolutionary algorithm based on the Non-dominated Sorted Genetic Algorithm-II (NSGA-II) [62,68] is utilised and seeks optimal solutions within the design space shown in Table 2. Table 3 demonstrates the five ‘candidate design points’ that have been evaluated by DesignXplorer [62] for the optimisation criteria stated in Table 1. Firstly, it can be seen that candidate design points include both single, double, and triple ridges. It is also evident that the design points are concentrated around specific areas of the design space. For instance, the geometry of the elliptical ridge has an HoW ratio of approximately 1.3≤HoW≤2.3, a CSR between 7% and 15%, and a pitch in the range of 1.9≤φ≤2.5. Finally, the length ratio is in the range of $51\%\leq L/L_{0}\leq 82\%$.

To assess the effectiveness of the newly proposed design points, a comparison is made against a smooth reference graft (i.e., one without any ridge). It is shown that the improvement in graft performance in terms of several key haemodynamic metrics is significant. In particular, it is observed that the optimised graft design with a helical ridge successfully induces a helical component in the flow field along with an increased WSS field. The increase in WSS is up to 24% higher than the reference case, while the area of abnormal WSS (WSSA < 1 Pa) is reduced by up to 76%. The helicity is found to be up to 3.3 times higher than the reference, while the recirculation area is in some cases eliminated. Finally, the WSSG is reduced by up to 46% compared to the reference case.

A significant trade-off is observed in all the candidate design points, which are found to induce an increased pressure drop compared with the reference graft of up to 87%. This observation is somewhat expected due to the increased resistance of the flow caused by the introduction of an occlusion, which directly affects the pressure drop along the graft. However, it is important to highlight that the pressure drops in this study are significantly higher than in some of the previous studies by the present authors (e.g., [26,28]). This is mainly because in all cases studied here, the ridge(s) are defined as having the same profile throughout their entire length. Therefore, the leading and trailing edges/faces of the ridge would have a flat/blunt surface, resulting in significant pressure loss. In reality, such faces (particularly the leading edge) are designed to have a gradual transitional shape (i.e., be more aerodynamic). In the present study, introducing such modifications to the leading/trailing surfaces would have resulted in inconsistencies when it came to optimisation. Nevertheless, keeping in mind this drawback, it should be noted that the overall impact of the helical ridge is positive for the haemodynamic environment of the flow and should be considered a favourable solution compared with the simple graft without ridges.

**Figure 2:**
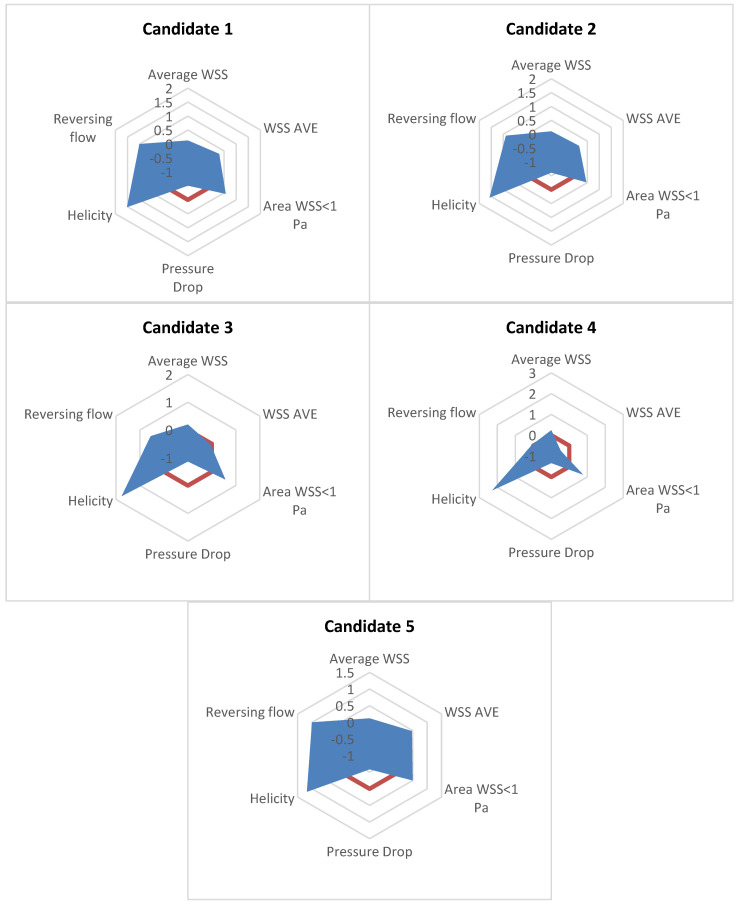
Comparison of the different candidates’ performances compared with the reference case with no ridge (red line): positive values denote improved performance compared with the reference solution. Please refer to Table 4 for the scale/numbers shown in each figure.

To identify the optimal design among the proposed candidate points found by the evolutionary algorithm used in the present study, the performance of each design point is compared with the rest. In Table 3, it is shown that candidate designs 1, 2, and 5 manage to perform better than the reference (no-ridge) graft for every optimisation criterion (as described in Section 3) except for the pressure drop. On the other hand, candidates 3 and 4 show the best performance amongst the candidates in the average WSS, helicity, and abnormal WSSA (<1 Pa), but they manage the higher reversing flow portion and a WSSG that even exceeds the one calculated for the reference. Figure 2 and Table 4 also quantify the performance of the candidates when compared with the reference solution of the graft with no ridges. Table 4 shows the area measured in Figure 2 normalised with the area of the reference graft with no ridges. Note that in Figure 2, positive values denote an improved performance compared with the reference results. Therefore, the larger the area of the candidate designs in Figure 2, the better the performance compared with the reference solution. From Table 4, it is again shown that Candidate 1 has the best performance amongst the five candidate designs. From all the above, Candidate 1 is chosen as the optimal graft design for the examined design space.

### 3.3. Assessment of the Optimised Design with Reference Geometries

#### 3.3.1. Steady-State Simulations

To evaluate the scale of improvement of the proposed geometry, a comparison is made between the smooth graft used in Section 3.2 and a single ridge graft design with the geometric characteristics proposed by Kabinejadian et al. [28], which is believed to resemble the geometry of the “Spiral Laminar Flow” (SLF) peripheral vascular graft commercialised by Vascular Flow Technologies (VFT) Ltd., Dundee, UK. As shown in Table 5, there is a clear progression from the no-ridge reference design to the baseline single ridge of [28] and the optimised design of the current study. Although most of the haemodynamic parameters are improved with the single ridge design of [28], there is an even further improvement offered by the design of the current work. Specifically, although the baseline ridge design is found to increase the WSS and reduce the WSSA < 1 Pa, the optimised design is found to further improve these results by at least a further 10%. The progression between the various designs of the helicity measure is also distinctive, where the baseline single-ridge design offers a 34% improvement compared with the reference, and the optimised design of the current work improves this measure by a further 88%. Regarding the reversing flow area and the pressure drop, the optimised design shows a marginal difference from the baseline ridge design. This highlights the added benefit of using the optimised geometry: for similar flow resistance, the optimised design manages to induce a much improved haemodynamic environment.

**Figure 3:**
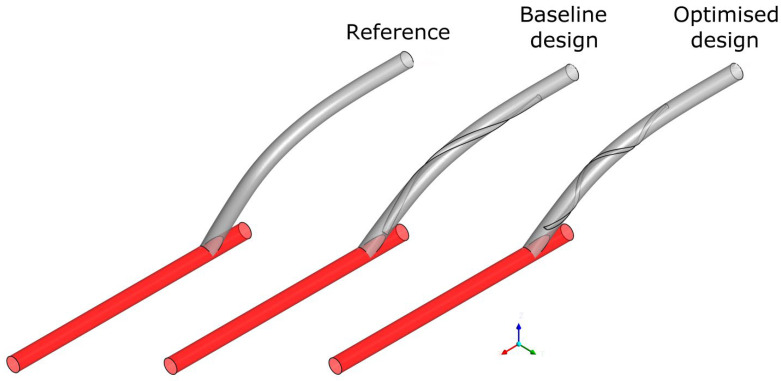
Comparison of the configuration of the reference, the baseline ridge design, and the optimised results of the current work.

**Figure 4:**
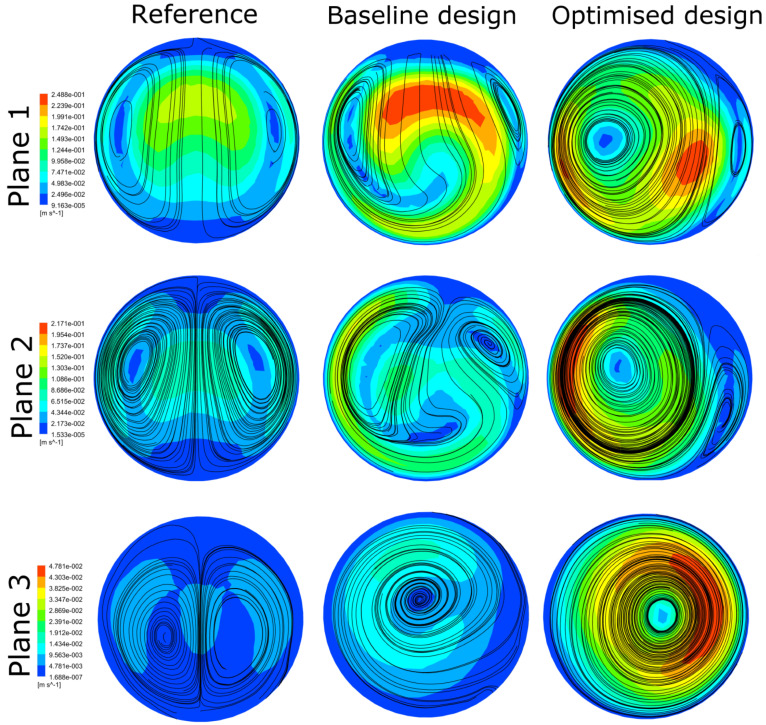
Secondary velocity profiles in three monitoring planes positioned 2 mm, 6 mm, and 50 mm after the anastomosis.

**Figure 5:**
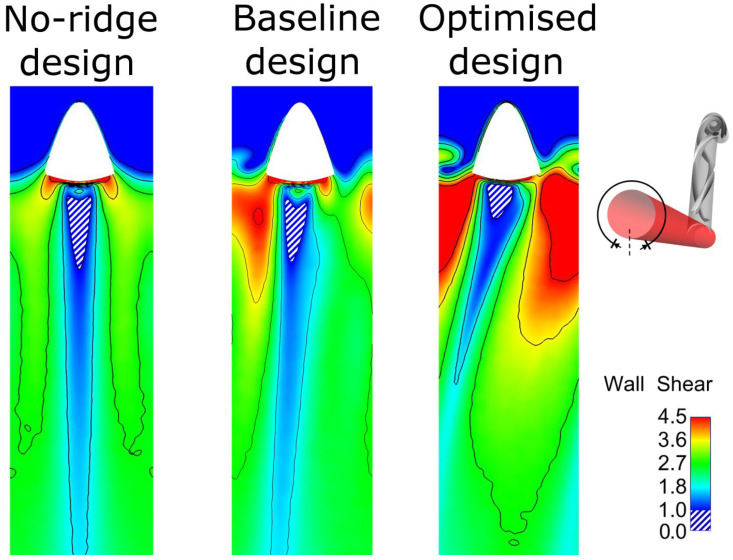
Wall shear stress contours on the arterial wall projected on a flat surface: the area near the graft anastomosis with WSS < 1 Pa has been marked with blue and white stripes.

**Figure 6:**
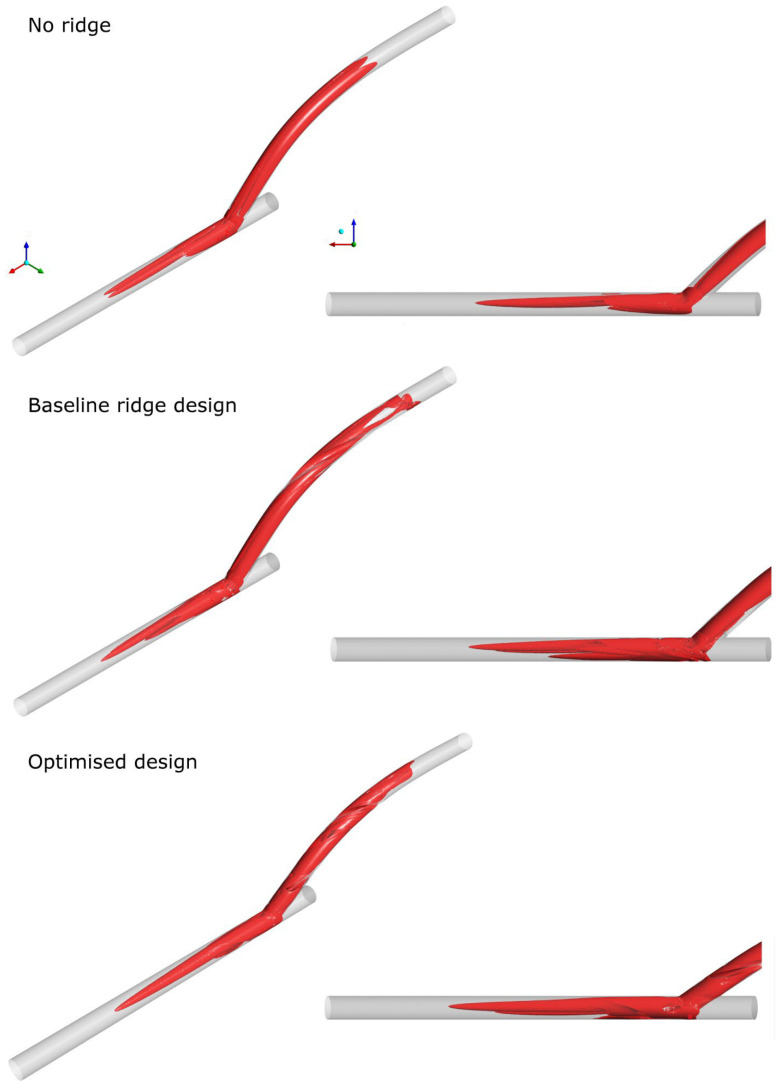
Helicity isosurfaces for the reference, baseline ridge design, and optimised design of the current work.

Figure 3 illustrates the different configurations of the three geometries: the reference without any ridges, the baseline ridge design [28], and the optimised design of the current work. The major differences shown between the two designs with a helical ridge are the reduced length and occlusion area of the ridge and the increased pitch for the optimised design (shown qualitatively in Table 5). These have the effect of reducing the graft’s obstruction to the flow, combined with an improved haemodynamic environment in the host artery. Figure 4 and Figure 5 offer a further insight into the benefits of performance gained by the optimised design as compared to the other two references. As shown, the secondary velocity field has a far higher measure in the current optimised design compared with its predecessors. This has the effect of minimising any areas of stagnating flow, as discussed in detail in the literature [19,20,21,22,23,24]. This observation is further supported by Figure 6, where the isosurfaces of high helical intensity are shown. As observed with the optimised design, the flow retains a helical environment much further downstream compared with the other two designs. Additionally, the helical intensity is significantly stronger with the optimised design. Figure 5 shows the comparison of the WSS on the host artery projected as a plane view (using Ensight©, CEI Inc., Research Triangle Park, NC, USA). It is shown clearly that the optimised design manages to improve the distribution of the WSS values while minimising the area where abnormal WSS values appear (WSSA < 1 Pa). As a result, most of the surface of the host artery experiences normal WSS conditions when the optimised design is used.

#### 3.3.2. Assessment of Optimised Geometry in Transient Simulations

Having assessed the optimised design of the helical graft against reference cases using common haemodynamic metrics, such as the WSS, WSSG, helicity, and recirculation area, as presented in Section 2.2, in the current section, advanced WSS-based metrics are used to gain a further insight into the optimised graft’s performance. These include the Time-Averaged WSS (TAWSS) [77], Oscillatory Shear Index (OSI) [77], and Relative Residence Time (RRT) [78], calculated according to Equations (11)–(13):

(11) $$\mathrm{TAWSS}=\frac{1}{T}\int_{0}^{T}\left|{\vec{\tau }}_{W}\right|\mathrm{dt},$$

(12) $$\mathrm{OSI}=\frac{1}{2}\left(1-\frac{\left|\int_{0}^{T}{\vec{\tau }}_{W}\mathrm{dt}\right|}{\int_{0}^{T}\left|{\vec{\tau }}_{W}\right|\mathrm{dt}}\right),$$

(13) $$\mathrm{RRT}=\frac{1}{\left(1-2\times \mathrm{OSI}\right)\times \mathrm{TAWSS}}=\frac{1}{\frac{1}{T}\left|\int_{0}^{T}{\vec{\tau }}_{W}\mathrm{dt}\right|},$$

where ${\vec{\tau }}_{W}$ is the WSS vector and *T* is the time period of the flow cycle. These parameters have been chosen because (i) localised distributions of low-WSS and high-OSI strongly correlate with the locations of atheroma [77], (ii) platelet activation may be induced by the combination of long exposure times and high shear stress [79,80,81,82], and (iii) stagnant and recirculating flow regions can cause platelet aggregation and thrombogenesis [83]. Clearly, these metrics can offer a valuable insight into the graft’s performance and complement well with the results shown in Section 3.3.1. It should be noted here that despite the usefulness of these metrics, their implementation in GDO studies would have been cumbersome due to computational limitations.

**Figure 7:**
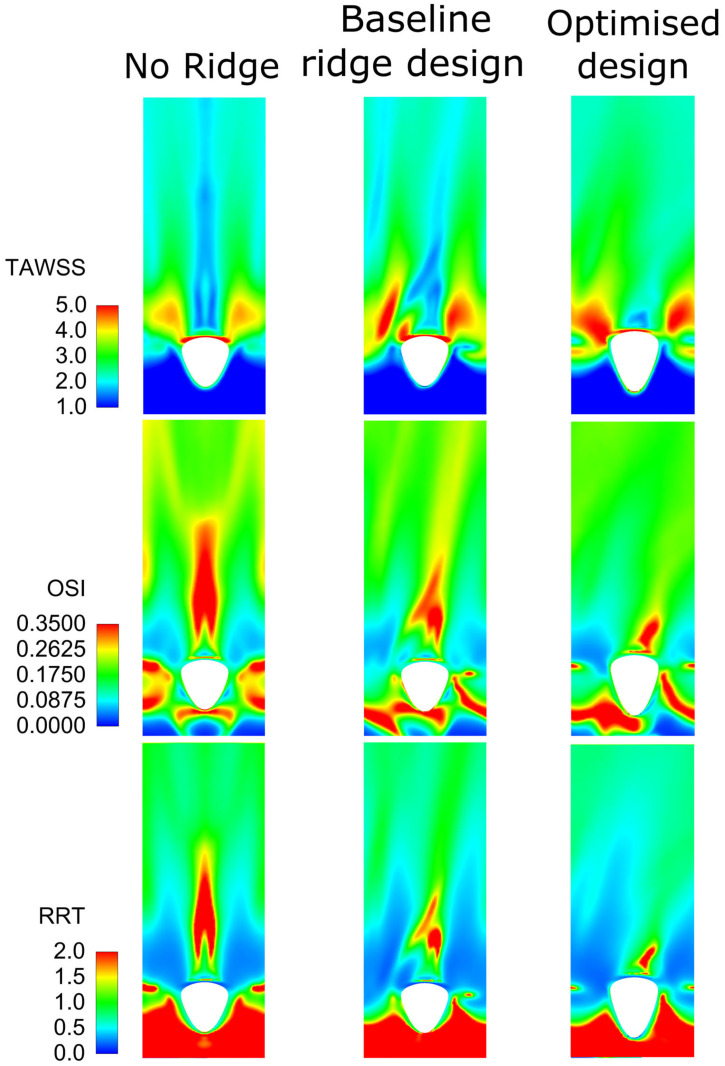
Wall shear stress derived metrics of TAWSS, OSI, and RRT for the reference, baseline ridge design, and optimised design of the current work.

Figure 7 shows the comparison between the three cases for TAWSS, OSI, and RRT as described in Equations (11)–(13). It is shown that although the baseline design improves the reference results with no ridge, with the optimised design the haemodynamic environment is further improved. Specifically, the area of increased TAWSS is much higher for the optimised design compared with the other two reference designs. Additionally, the areas that were increased in OSI and RRT are reduced with the introduction of a baseline ridge design and further minimised with the optimised geometry. From the above, it is deduced that the optimised design should decrease the danger of atheroma formation since the areas of low TAWSS and high OSI are less than those of the baseline and no-ridge designs [77]. Moreover, the reduced areas of RRT show a decreased danger of platelet activation and thrombogenesis [79,80,81,82]. It should be noted that the baseline design also improves the haemodynamic environment over the no-ridge design (as shown in [28]), but when compared with the optimised design, it is clearly found to be deficient.

From the above, it is clear that although the introduction of a ridge, as presented in [28], improves the flow environment compared with the no-ridge design, the design of the current work further improves the helical graft performance. While the above findings have pointed towards an optimal design of the bypass graft with a helical ridge, which significantly improves what is currently used in practice, further studies are necessary to fully assess the effect of this design in operational use. Computational limitations such as the boundary conditions and the steady-state assumption of the flow should be taken into account to understand if they play any part in the final results.

## 4. Conclusions

In the present work, a thorough multi-objective optimisation study has been carried out aiming to optimise the haemodynamics after the introduction of a bypass graft with a helical ridge. Careful consideration of the design space as well as the optimisation criteria, which are based on the widely accepted assumptions of normal values of WSS, low destruction, and disturbance of the flow, have led to a set of candidate design points with optimal characteristics to be proposed. Although minor drawbacks were inevitably found with the introduction of a helical ridge, it was shown that crucial haemodynamic characteristics are improved with the proposed design. The outcomes and the methodology followed in this study can be used to inform bypass graft designs with improved long-term performance and patency.

## Figures and Tables

**Table 1:** Optimisation criteria targets.

Optimisation Criteria	Target
Helicity	Maximise
WSS on host artery	Maximise
WSSA < 1 Pa on host artery	Minimise
WSSG on host artery	Minimise
Reversing a portion of the flow	Minimise
Pressure drop along the graft region	Minimise

**Table 2:** The range of design parameters before the parameter correlation study.

Design Parameter	Range
Ridge count	1–3
Ridge Elliptical Height/Width ratio	0.4–2.5
Cross-Sectional Ratio (CSR)	5–25
Trailing Edge Orientation (TEO)	$0{}^{\circ}–\frac{360{}^{\circ}}{RidgeCount}$
Pitch (φ) [turns/L]	0.5–3.0 [turns/L]
Ridge length ratio (*L*/*L*_0_)	25–100·L
Graft-artery anastomosis angle (θ)	30°−70°

**Table 3:** Candidate design points and their comparison to a reference solution (with no ridges). Note that (a) green shading and bold is the optimal value, (b) green shading is the second optimal value, (c) red shading and bold is the least optimal value, and (d) red shading is the second least optimal value. The difference from the reference solution is shown in the bracket.

Design Parameter	Candidate 1	Candidate 2	Candidate 3	Candidate 4	Candidate 5	Reference
Ridge count	1	1	2	2	3	-
Ridge Elliptical Height/Width ratio (HoW)	2.19	1.78	1.33	1.66	2.40	-
Cross-Sectional Ratio (CSR)	8.0	11.3	15.2	10.7	7.0	-
Ridge Orientation α(TEO)	332.9°	301.0°	61.6°	72.2°	110.4°	-
Pitch φ$\;[\mathrm{turns}/L_{0}$]	2.34	1.95	2.49	2.21	2.35	-
$\mathrm{Ridge}\;\mathrm{length}\;\mathrm{ratio}\;\left(L/L_{0}\right)$	73	82	58	52	68	-
Distal anastomosis angle (θ)	36°	35°	40°	64°	32°	45°
**Optimisation Criteria**						
Helicity [J kg^−1^]	1.57 $\left(+151.7\%\right)$	1.60 $\left(+157.3\%\right)$	1.72 $\left(+175.9\%\right)$	2.04 $\left(+227.7\%\right)$	1.36 $\left(+117.5\%\right)$	0.623
WSS on host artery [Pa]	2.39 $\left(+12.6\%\right)$	2.34 $\left(+10.4\%\right)$	2.54 $\left(+19.6\%\right)$	2.63 $\left(+23.9\%\right)$	2.37 $\left(+11.9\%\right)$	2.12
Area of WSS < 1 Pa on host artery [mm^2^]	10.0 $\left(-56.4\%\right)$	12.4 $\left(-46.0\%\right)$	10.1 $\left(-56.0\%\right)$	5.52 $\left(-76.0\%\right)$	11.6 $\left(-49.7\%\right)$	23.0
WSSG on host artery [kg m^−2^ s^−2^]	428.0 $\left(-29.3\%\right)$	509.5 $\left(-15.8\%\right)$	$638.7\;\left(+5.56\%\right)$	897.6 $\left(+48.3\%\right)$	321.8 $\left(-46.8\%\right)$	605.1
Reversing a portion of the flow	0 $\left(-100.0\%\right)$	0.45 $\left(-89.1\%\right)$	1.85 $\left(-55.2\%\right)$	3.83 $\left(-7.19\%\right)$	0 $\left(-100.0\%\right)$	4.1
Pressure drop along the graft region [Pa]	225.5 $\left(+51.2\%\right)$	240.6 $\left(+61.3\%\right)$	279.1 $\left(+87.1\%\right)$	248.9 $\left(+66.9\%\right)$	236.8 $\left(+58.8\%\right)$	149.14

**Table 4:** Comparison of the different candidates’ performances as compared with the reference (no-ridge) cases as in Figure 2.

Candidate Number	$\left\{\frac{\mathrm{Candidate}\;\mathrm{Area}}{\mathrm{Reference}\;\mathrm{Area}}\right\}\;$
1	2.13
2	1.91
3	1.55
4	1.34
5	1.99

**Table 5:** Comparison between ‘Candidate design 1’ (taken from Table 3) and the reference solution without any ridges and a helical graft with a single ridge with dimensions similar to the Vascular Flow Technologies (VFT) graft according to [28].

**Design Parameter**	**Reference**	**Single-Ridge Design [28]**		**Candidate 1**		
Ridge count	-	1		1		
Ridge Elliptical Height/Width ratio (HoW)	-	1.2		1.56		
Occlusion area (%)	-	10.4		8.3		
Ridge Orientation α (TEO)	-	180.0°		265.7°		
Pitch φ$\;[\mathrm{turns}/L_{0}$]	-	1.115		1.72		
$\mathrm{Ridge}\;\mathrm{length}\;\mathrm{ratio}\;L/L_{0}$	-	90		33.9		
Distal anastomosis angle θ	45°	45°		45.5°		
**Design Parameter**			**Ref.**		**Ref.**	**Single Ridge** [28]
WSS on host artery [Pa]	2.12	2.15	+1.5%	2.39	+12.6%	+10.9%
Area of WSS < 1 Pa on host artery [mm^2^]	23.0	15.6	−32.3%	10.0	−56.4%	−35.6%
WSSG on host artery [kg m^−2^ s^−2^]	605.0	665.2	+9.93%	428.0	−29.3%	−35.7%
Reversing a portion of the flow	4.13	4.9	+19.8%	0	−100.0%	−100.0%
Helicity [J kg^−1^]	0.623	0.834	+33.9%	1.57	+151.7%	+88.0%
Pressure drop along the end anastomosis [Pa]	149.1	229.3	+53.8%	225.5	+51.2%	−1.68%

## Data Availability

Not applicable.
